# Disruption of the Microglial ADP Receptor P2Y_13_ Enhances Adult Hippocampal Neurogenesis

**DOI:** 10.3389/fncel.2018.00134

**Published:** 2018-05-17

**Authors:** Jennifer Stefani, Olga Tschesnokowa, Marta Parrilla, Bernard Robaye, Jean-Marie Boeynaems, Amparo Acker-Palmer, Herbert Zimmermann, Kristine Gampe

**Affiliations:** ^1^Institute of Cell Biology and Neuroscience and Buchmann Institute for Molecular Life Sciences, Goethe-University, Frankfurt am Main, Germany; ^2^Max-Planck-Institute for Brain Research, Frankfurt am Main, Germany; ^3^Institute of Interdisciplinary Research, School of Medicine, Université Libre de Bruxelles, Bruxelles, Belgium; ^4^Focus Program Translational Neurosciences (FTN), University of Mainz, Mainz, Germany

**Keywords:** adult neurogenesis, ADP, ATP, dentate gyrus, hippocampus, microglia, P2Y_13_ receptor, progenitor cell

## Abstract

In mammalian species, including humans, the hippocampal dentate gyrus (DG) is a primary region of adult neurogenesis. Aberrant adult hippocampal neurogenesis is associated with neurological pathologies. Understanding the cellular mechanisms controlling adult hippocampal neurogenesis is expected to open new therapeutic strategies for mental disorders. Microglia is intimately associated with neural progenitor cells in the hippocampal DG and has been implicated, under varying experimental conditions, in the control of the proliferation, differentiation and survival of neural precursor cells. But the underlying mechanisms remain poorly defined. Using fluorescent *in situ* hybridization we show that microglia in brain express the ADP-activated P2Y_13_ receptor under basal conditions and that *P2ry13* mRNA is absent from neurons, astrocytes, and neural progenitor cells. Disrupting *P2ry13* decreases structural complexity of microglia in the hippocampal subgranular zone (SGZ). But it increases progenitor cell proliferation and new neuron formation. Our data suggest that P2Y_13_ receptor-activated microglia constitutively attenuate hippocampal neurogenesis. This identifies a signaling pathway whereby microglia, via a nucleotide-mediated mechanism, contribute to the homeostatic control of adult hippocampal neurogenesis. Selective P2Y_13_R antagonists could boost neurogenesis in pathological conditions associated with impaired hippocampal neurogenesis.

## Introduction

Microglia have long been known as major orchestrators of the brain inflammatory response. They actively move to sites of damage and engulf and eliminate neural debris. But microglia also contribute to physiological brain functions. They continuously survey their cellular environment and regulate neuronal survival, migration of neurons, axonal growth, pruning of supernumerary synapses, and functional maturation of developing synapses (Nayak et al., 2014; Sierra et al., 2014). The role of microglia in the control of developmental and adult neuron formation has recently received increasing attention (Gemma and Bachstetter, 2013). Microglia is intimately associated with neural progenitor cells in the adult hippocampal dentate gyrus (DG) and has been implicated in the control of neurogenesis. But the underlying mechanisms remain poorly defined.

In mammalian species, including humans, the hippocampal DG is a primary region of adult neurogenesis (Frisén, 2016). Hippocampal neurogenesis confers an additional level of plasticity to the adult brain and has been implicated in memory consolidation, pattern separation, and mood regulation but also in regulating “forgetting” (Christian et al., 2014). Moreover, aberrant adult hippocampal neurogenesis is associated with neurological pathologies. Understanding the molecular mechanisms controlling adult hippocampal neurogenesis is expected to open new therapeutic strategies for mental disorders such as depression or schizophrenia (Jun et al., 2012). Multiple evidence suggests that new neuron formation in the DG is initiated by the activation of astroglia-like type 1 cells whose cell bodies are located in the subgranular zone (SGZ), followed by several intimately apposed transient functional cellular stages, which can be addressed by specific marker profiles. Type 1 cells give rise to highly proliferating intermediate progenitor cells (type 2 cells) enlarging the neurogenic pool. The next stage comprises fate specification and the formation of type 3 cells (neuroblasts). These exhibit little proliferating activity and give rise to mature excitatory principal neurons that become incorporated into the granule cell layer (GCL; Kempermann et al., 2015).

Neurogenesis needs to be under strict homeostatic control, tightly balancing new neuron formation via proneurogenic mechanisms and neurogenesis-attenuating mechanisms. These include the control of cell proliferation, elimination of surplus cells, and new neuron formation. A considerable variety of external signaling cues, including growth factors, neurotrophic factors, and neurotransmitters as well as intracellular signaling pathways and epigenetic regulators have been implicated in the control of adult hippocampal neurogenesis. Factors may be derived from the neurogenic niche including its vasculature or from axonal inputs (Aimone et al., 2014; Gonçalves et al., 2016; Yao et al., 2016). Increasing evidence suggests that extracellular nucleotides contribute to the control of both embryonic and adult neurogenesis (Lecca et al., 2016; Oliveira et al., 2016). They activate specific receptors representing either Na^+^, K^+^ and Ca^2+^ permeable ion channels (seven P2X receptors) or G protein-coupled receptors (in rodents seven P2Y receptors). Nucleotides function as primary messengers in intercellular communication and can stimulate the release of other extracellular messenger substances. Depending on receptor subtype, these include hormones, neurotransmitters, growth factors, a considerable variety of other proteins including enzymes, numerous cytokines, lipid mediators, nitric oxide, and reactive oxygen species. Moreover, nucleotides activate or co-activate growth factor receptors (Zimmermann, 2016).

Microglia express a variety of receptors for both nucleotides and adenosine (Koizumi et al., 2013) and the extracellular nucleotide-hydrolyzing enzyme nucleoside triphosphate diphosphohydrolase 1 (NTPDase1; Braun et al., 2000; Färber et al., 2008; Matyash et al., 2017), which catalyzes the dephosphorylation of nucleoside triphosphates and diphosphates to the nucleoside monophosphates, terminating their effects on nearby nucleotide receptors. In contrast to the *in situ* situation, essentially all P2 receptors are expressed by cultured microglia (Bianco et al., 2005). Moreover, culturing greatly alters microglial P2 receptor expression (Crain et al., 2009), suggesting that *in situ* analyses are essential for evaluating the implication of P2 receptors in microglial function. Nucleotide receptors expressed by microglia *in situ* include the ATP-activated P2X7 and P2X4 receptors which are strongly upregulated under diverse pathological conditions, the G_q_-coupled and UDP-activated P2Y_6_ receptor (P2Y_6_R) and three closely related G_i_-coupled receptors, the ADP-activated receptors P2Y_12_ (P2Y_12_R) and P2Y_13_ (P2Y_13_R), and the UDP-glucose/UDP-activated P2Y_14_ receptor (P2Y_14_R). The P2Y_6_R has been implicated in microglial phagocytosis (Koizumi et al., 2007) and the P2Y_12_R in mediating rapid microglial chemotaxis at early stages of the response to local CNS injury (Haynes et al., 2006).

The more recently characterized P2Y_13_R (Communi et al., 2001; Zhang et al., 2002) is expressed in several tissues, including spleen, bone, liver, pancreas, and heart, or also in peripheral leukocytes (Pérez-Sen et al., 2017). *P2ry13* KO mice exhibit a small increase in bone area but no other major abnormalities. Body weight, fat mass, and lean body mass are normal. Hepatic high-density lipoprotein (HDL) cholesterol uptake and biliary cholesterol content and output were found to be decreased. But their plasma HDL levels and other lipid levels were described as normal or only slightly decreased (Blom et al., 2010; Fabre et al., 2010). The P2Y_13_R is also expressed by osteoblasts and involved in osteogenesis. Studies on *P2ry13* KO mice reveal a decreased bone turnover associated with a reduction in the number of osteoblasts and osteoclasts at the bone surface (Wang et al., 2012) and an impact of the receptor on the balance of the terminal differentiation of bone marrow progenitors into osteoblasts and adipocytes (Biver et al., 2013).

Expression of the P2Y_13_R in cultured neurons (Miras-Portugal et al., 2016), cultured astroglia (Carrasquero et al., 2009) and spinal cord microglia *in situ* (Kobayashi et al., 2012) has been reported. After peripheral nerve injury the P2Y_13_R is upregulated in spinal cord microglia together with the P2Y_6_R, the P2Y_12_R, and the P2Y_14_R (Kobayashi et al., 2012) and may be involved in the induction and maintenance of neuropathic pain (Tatsumi et al., 2015). Otherwise functional roles of the P2Y_13_R or of the P2Y_14_R in the central nervous system *in situ* are unknown. Importantly, the impact of the P2Y_13_R may have been overlooked in previous studies targeting the P2Y_12_R and using ligands that are now known to antagonize both the P2Y_12_ and P2Y_13_R (2-methylthio-AMP and AR-C69931MX).

In this study we determined the cellular expression of the P2Y_13_R by fluorescent *in situ* hybridization (FISH). We then elucidated the functional role of the P2Y_13_R in hippocampal neurogenesis *in situ* under basal conditions using the *P2ry13* null mouse model (Fabre et al., 2010). Our data locate the P2Y_13_R to hippocampal microglia and imply that it supports structural complexity of microglia and constitutively attenuates neural progenitor cell proliferation. This identifies a signaling pathway whereby microglia via a nucleotide-mediated mechanism contribute to the homeostatic control of adult hippocampal neurogenesis.

## Materials and Methods

### Animals

All animal experiments were conducted according to the institutional guidelines, approved by the Animal Research Board of the State of Hesse (Regierungspraesidium Darmstadt) and conducted under veterinary supervision in accordance with European regulations. *P2ry13* KO mice (Fabre et al., 2010) and corresponding C57BL/6 WT mice were bred in house. To ease the identification of primary neural stem cells in the hippocampal neurogenic niche we crossed mice expressing the enhanced green fluorescent protein (GFP) under the control of the nestin promoter (kindly provided by Grigori Enikolopov, Cold Spring Harbor Laboratory; Mignone et al., 2004) with *P2ry13* KO mice. Nestin-driven EGFP expression was confirmed by genotyping 3–4 week old mice using oligonucleotides. Mice of two different age groups were analyzed, young adult mice (8–12 weeks) and aged mice (20–24 weeks). For immunocytochemical analysis animals received an anesthetic overdose by intraperitoneal injection of ketamine (180 mg/kg of body weight; Ketavet) and xylazine (10 mg/kg of body weight; Rompun) and were intracardially perfused with 10 ml of ice-cold physiological saline (0.9% NaCl) followed by perfusion with 150 ml ice-cold 4% paraformaldehyde in phosphate-buffered saline (PBS: 137 mM NaCl, 2.7 mM KCl, 10.1 mM Na_2_HPO_4_, 1.8 mM KH_2_PO_4_, pH 7.4). Removed brains were postfixed over night at 4°C in the same fixative and cryoprotected with 30% sucrose/PBS for 24–48 h at 4°C.

### Immunohistochemistry

For standard immunostaining, antigen retrieval was performed by incubating sections in sodium citrate buffer (10 mM sodium citrate, 0.05% Tween 20, pH 6.0) at 80°C for 30 min. Following several rinses in PBS, sections were blocked with 5% bovine serum albumin (BSA) in 0.5% Triton X-100/PBS for 1 h at room temperature. The following primary antibodies (in 1% BSA + 0.5% Triton X-100/PBS) were applied overnight at 4°C: anti-Ki-67 (M7249, 1:5, Dako Cytomation), anti-Tbr2 (ab183991, 1:200, Abcam), anti-doublecortin (DCX; sc-8066, 1:200, Santa Cruz Biotechnology), anti-cleaved caspase-3 (Asp175; 9661, 1:500, Cell Signaling Technology), anti-glial fibrillary acidic protein (GFAP; G-3893, 1:500 and G-9269, 1:500, both Sigma-Aldrich), anti-GFP (ab13970, 1:500, Abcam), anti-ionized calcium binding adaptor molecule 1 (Iba1; 019-19741, 1:500, Wako Chemicals). Sections incubated with the secondary antibodies Alexa Fluor 488-coupled donkey anti-chicken (703-546-155), Cy3-coupled donkey anti-goat (705-165-147), Cy3-coupled donkey anti-rabbit (711-165-152), Cy5-coupled donkey anti-mouse (715-175-151) (all 1:200 and from Dianova), and Alexa Fluor 488-coupled donkey anti-rabbit (A-21206), Alexa Fluor 647-coupled donkey anti-goat (A-21447; both 1:200 and from Molecular Probes), or Alexa Fluor 488-coupled donkey anti-rabbit (A21206), Alexa Fluor 647-coupled donkey anti-goat (A21447), Alexa Fluor 594-coupled donkey anti-rat (A21209; all 1:200 and from Life Technologies), and with DAPI were mounted onto glass slides.

In case of immunoperoxidase detection, sections were pretreated with 0.6% H_2_O_2_/PBS before blocking with 5% BSA in 0.05% Triton X-100/PBS and incubated with the anti-Iba1 antibody in the same blocking solution (overnight at 4°C). Following several rinses in PBS, sections were incubated for 2 h with biotin-coupled goat anti-rabbit secondary antibody (1:1000, B7389, Sigma) and an avidin-biotin-peroxidase complex (Vector Laboratories) was applied for 2 h. Sections were subsequently incubated in diaminobenzidine (DAB) substrate solution (SIGMAFAST™ DAB with Metal Enhancer, Sigma-Aldrich) according to the manufacturer’s protocol, dehydrated in graded ethanol, and mounted with Roti-Histokit II (Carl Roth).

For detection of BrdU labeling in the DG, floating cryosections were incubated in 50% formamide in 2× saline-sodium citrate buffer (SSC: 0.3 M NaCl, 0.03 M sodium citrate, pH 7.0) at 65°C for 2 h and rinsed in 2× SSC followed by 30 min incubation in 2 M HCl at 37°C. Sections were neutralized in 0.1 M boric acid (pH 8.5) for 10 min, washed in Tris-buffered saline (TBS: 0.15 M NaCl, 0.1 M Tris-HCl, pH 7.5) and blocked with 3% normal donkey serum in TBS containing 0.1% Triton-X-100 for 30 min at room temperature, followed by incubation with an anti-BrdU antibody (1:500, OBT0030, AbD Serotec) in the same blocking solution (overnight at 4°C). For double immunostaining for BrdU and the neuronal marker neuronal nuclei (NeuN), an additional antibody (1:500, MAB377, Merck Millipore) was added. Following several rinses in TBS, sections were incubated with secondary antibodies (Cy3-coupled donkey anti-rat, 711-165-153, 1:200, Jackson Immune Research; Alexa Fluor 488-coupled donkey anti-mouse (A-21202, 1:200, Molecular Probes) and DAPI (4′,6-diamidino-2-phenylindole, 1:1000, 28718-90-3, Sigma-Aldrich) for 2 h at room temperature. Cryosections were washed in TBS and mounted in Aqua-Poly/Mount (Polysciences Europe).

### Fluorescent *in Situ* Hybridization

For characterizing the expression of *P2ry13* single- or two-color FISH was combined with immunohistochemistry. Young adult mice were anesthetized as described above, perfused with 20 ml of ice-cold 4% paraformaldehyde in PBS and dissected brains were postfixed overnight in the same fixative solution at 4°C. Brains were cryoprotected with increasing concentrations of sucrose in PBS (15%–30%), embedded in TissueTek and frozen. 16 μm thick coronal sections were cut with a cryostat (1 in 20 series). The riboprobes applied for *P2ry13* were F: ACAGACAACATCACCCTAGCCT, R: CCT CTCTTCTGGTGCTCTGTTT (corresponding to nucleotide positions 1055-1663 [accession number NM_028808.3, available from Allen Bain Atlas]^1^ and for *Cx3cr1* F: GACGATT CTGCTGAGGCCTGTTA, R: CCTCGCTTGTGTAGTGAGTG AAAC (corresponding to nucleotide position 147-1143) [accession number NM_028808.3]). Riboprobes were designed and synthesized as previously described (Ishii et al., 2004; Parrilla et al., 2016) using hippocampus cDNA as a template. Single- or two-color FISH combined with immunohistochemistry was performed as described (Ishii et al., 2004) with slight modifications: (1) hybridization step: slices were incubated in 12 μg/ml of proteinase K solution at 37°C for 16 min; (2) hybridization of *P2ry13* and *Cx3cr1* riboprobes labeled with digoxigenin and fluorescein, respectively, was performed at 65°C overnight in the hybridization solution (Parrilla et al., 2016); and (3) detection step: antibodies against digoxigenin-alkaline phosphatase (11093274910, Roche) and fluorescein horseradish peroxidase (NEF710, PerkinElmer) were incubated together with the following primary antibodies: anti-Iba1, anti-GFAP, anti-SRY (sex determining region Y)-box 2 (Sox2; Sc-17320, 1:200, Santa Cruz), anti-DCX, anti-NeuN (ABN78, 1:500, Millipore), anti-somatostatin (AB5494, 1:200, Millipore) and/or anti-parvalbumin (PV27, 1:200, Swant). Signals of digoxigenin and fluorescein probes were detected as described (Parrilla et al., 2016). Primary antibodies were detected with Alexa Fluor 488-coupled donkey anti-mouse (A21202, 1:500), donkey anti-rat (A21208, 1:500) or Alexa Fluor 647-coupled donkey anti-rabbit (A31573, 1:250) or donkey anti-goat (A21447, 1:250) secondary antibodies (all from Life Technologies). Images are a maximal projection of a z-stack group of pictures taken with a Leica TCS SP5 II confocal microscope. XZ and YZ images were obtained with the orthogonal sectioning tool from Leica Microsystems LAS AF software using their corresponding z stack group of pictures as templates. Brightness and contrast of the images were adjusted with Adobe Photoshop CS6 software. Each analysis was repeated in three animals.

The degree of colocalization of immunolabeling for Iba1 and the FISH signal for *P2ry13* mRNA was analyzed using the maximal projection of a z-stack group of images of coronal sections of the DG (16 μm) taken with an objective of 25× of three WT animals (at least four coronal sections each). The total numbers of *P2ry13*^+^ cells per animal were 312, 127 and 338, and the number of cells colocalizing with Iba1 were 293, 116 and 320, respectively. The percentage colocalization was calculated for each animal and the data are presented as mean ± SEM.

### Proliferation Analysis of Hippocampal Progenitor Cells by BrdU-Pulse Labeling

Young adult WT and *P2ry13* KO mice received either one single intraperitoneal injection of the thymidine analog 5-bromo-2’-deoxyuridine (BrdU; 50 mg/kg of bodyweight, Sigma-Aldrich) and were perfused 2 h after the final BrdU pulse or they were injected three times a day at 2 h interval with the same BrdU concentration and perfused either 3 days or 28 days after the last BrdU pulse (compare Figure 4A). Aged mice received either three BrdU pulses (50 mg/kg of bodyweight) at 2 h interval and were perfused 2 h after the final BrdU pulse or they received three daily BrdU pulses (150 mg/kg of body weight) at 2 h intervals on three consecutive days and were perfused 28 days after the final BrdU pulse (compare Figure 4I). Brains were frozen and serially cut (1 in 12 series) into free-floating sections, either 40 μm thick sagittal (young adult mice, from medial to lateral 2.725 mm to 0.225 mm, according to Allen Mouse Brain Atlas, 14 sections per animal, *n* = 7) or 50 μm thick coronal (aged mice, bregma −1.355 mm to −2.555 mm, according to Allen Mouse Brain Atlas, eight sections per animal, *n* = 7; Leica cryotome CM 3050 S, Leica Biosystems). Throughout the manuscript identical exposure times and parameters were chosen for sections from WT and *P2ry13* KO animals. All quantification procedures were performed in a double-blinded manner.

**Figure 1:**
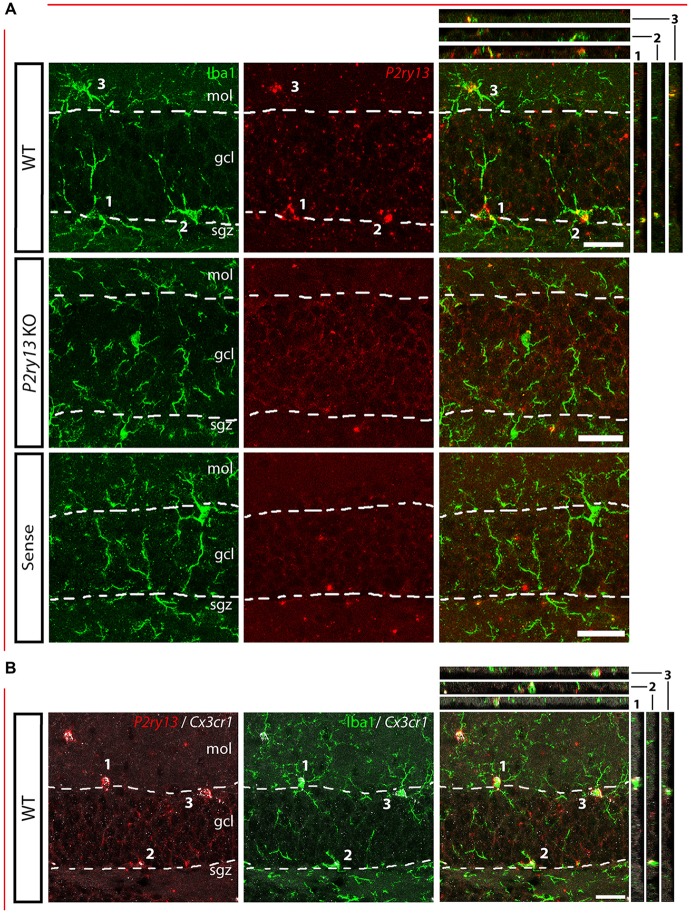
*P2ry13* mRNA is colocalized with microglia in the dentate gyrus (DG; young adults). **(A)** Details of DG, immunolabeling for Iba1 (green) and FISH for *P2ry13* (red), with overlay (right). Absence of FISH labeling in *P2ry13* KO mice and on application of the sense riboprobe for *P2ry13*. The granule cell layer (GCL) is outlined with dashed lines. Minor precipitates of the FISH reaction product occur in both sections from WT and *P2ry13* KO mice. **(B)** FISH for *Cx3cr1* as an alternative marker for microglia (WT tissue). Left: double FISH for *P2ry13* (red) and *Cx3cr1* (white); middle: immunolabeling for Iba1 (green) and FISH for *Cx3cr1* (white); right: merger of the images on the left and in the middle. Confocal image stacks; Colocalization is visualized in the right panels along the xz-axis (strips on the right) and yz-axis (strips on top) with the imaged cell bodies numbered 1–3. gcl, granule cell layer; mol, molecular layer; sgz, subgranular zone. Scale bars, 25 μm.

**Figure 2:**
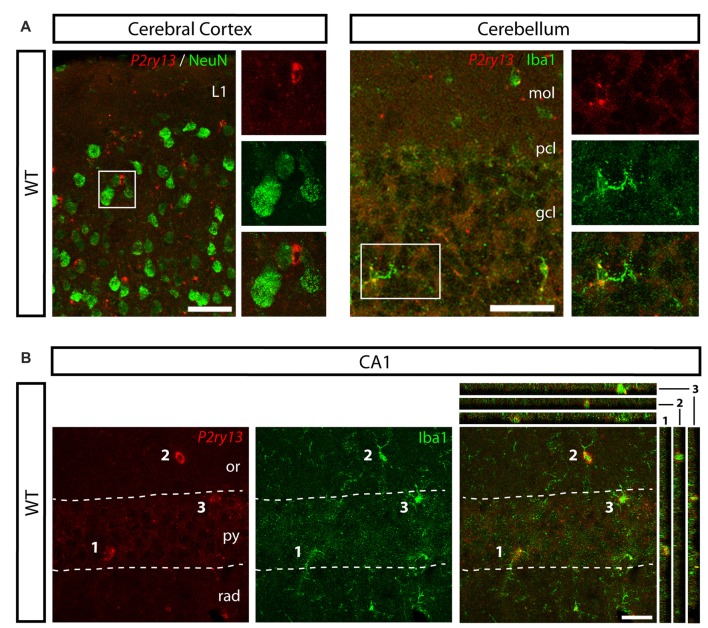
*P2ry13* mRNA is located in microglia in cerebral cortex, cerebellum and hippocampal CA1 region (young adults). **(A)** Absence of *P2ry13* mRNA from neurons in cerebral cortex (left) and cerebellum (right). Double labeling for *P2ry13* mRNA (red) and NeuN (green; cerebral cortex) or Iba1 (green; cerebellum). Close-ups of boxed images on the right with *P2ry13* mRNA (top), Iba1 (middle) and overlay (bottom). **(B)** Double labeling for *P2ry13* mRNA (red) and Iba1 (green), merger on the right, in the hippocampal CA1 region. Confocal image stacks; Colocalization is visualized in the right panel along the xz-axis (strips on the right) and yz-axis (strips on top) with the imaged cell bodies numbered 1–3. gcl, granule cell layer; mol, molecular layer; or, oriens layer; pcl, Purkinje cell layer; py, pyramidal cell layer; rad, radiant layer. Scale bars, 50 μm in **(A)**; 25 μm in **(B)**.

**Figure 3:**
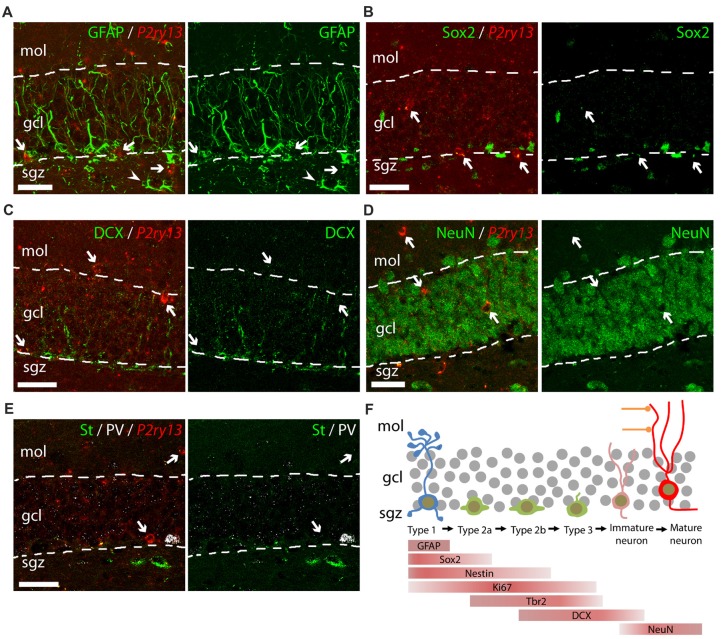
Lack of colocalization of *P2ry13* mRNA with other cell types of the DG (young adults). **(A–E)** Details of DG showing double labeling using FISH for *P2ry13* (red) and immunocytochemistry for various cell markers (green); overlay (left), immunolabeling (right; all WT animals). **(A)** Glial fibrillary acidic protein (GFAP), **(B)** Sox2, **(C)** DCX (weak immunolabeling due to experimental conditions required for FISH) **(D)** NeuN. **(E)** Triple labeling for somatostatin (green, St), parvalbumin (white PV) and for *P2ry13* (red). The GCL is outlined with dashed lines. Arrows, cell bodies labeled for *P2ry13* mRNA; arrow head, *P2ry13*-negative astrocyte. **(F)** Simplified schematic of principal cell types along the hippocampal neurogenesis pathway. Lineage markers used in this study and their cellular location are indicated by bars. gcl, granule cell layer; mol, molecular layer; sgz, subgranular zone. Scale bars, 25 μm.

**Figure 4:**
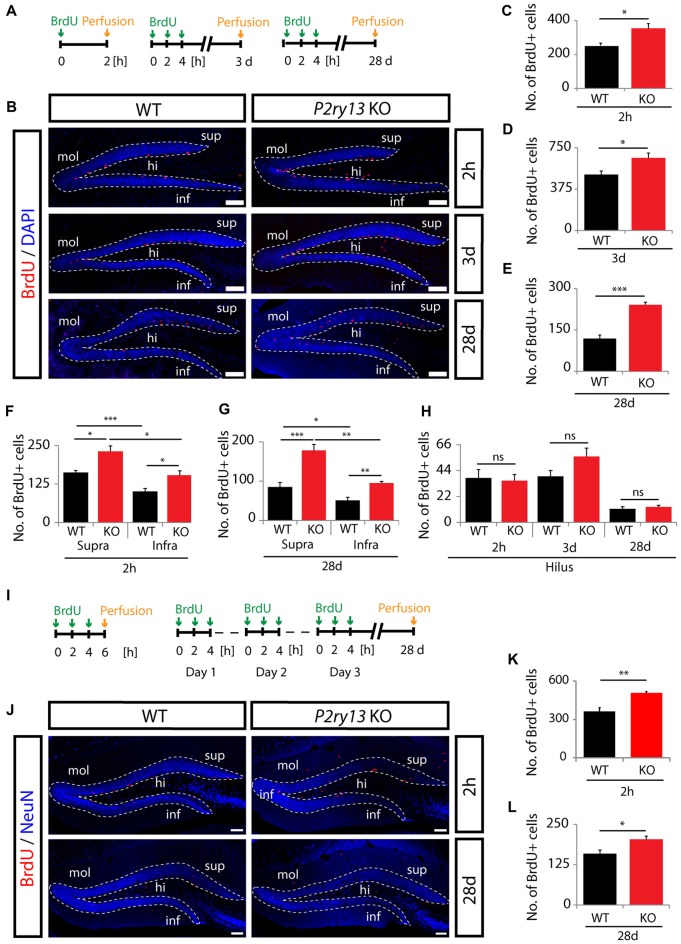
Increased precursor cell proliferation and long-term survival in young adult and aged *P2ry13* KO mice. **(A)** Schematic of the BrdU injection protocols in young adult mice. **(B)** BrdU (red) and DAPI staining (blue) in the DG (dashed lines) of adult WT and *P2ry13* KO mice 2 h, 3 days or 28 days after BrdU injection. **(C–E)** Total number of BrdU^+^ cells in young adult dentate gyri (14 corresponding sagittal sections per animal, *n* = 7). **(C)** Corresponding to upper panel in **(B)** 2 h after BrdU injection. **(D)** Corresponding to middle panel in **(B)** 3 days after BrdU injection. **(E)** Corresponding to lower panel in **(B)** 28 days after BrdU injection. **(F)** Total number of BrdU^+^ cells in the hilar region at the time points shown (corresponding to **C–E**). **(G,H)** Separate quantification of total numbers of BrdU^+^ cells in the suprapyramidal and infrapyramidal layer of the DG corresponding to **(C,E)**, respectively. **(I–L)** Separate series of experiments using aged mice. **(I)** Schematic of the BrdU injection protocols. **(J)** Staining for BrdU (red) and NeuN (blue) in the DG (dashed lines) of aged mice 2 h (upper panel) and 28 days (lower panel) after the final BrdU pulse. **(K,L)** Total number of BrdU^+^ cells in aged mice (eight corresponding coronal sections per animal, *n* = 7). **(K)** Corresponding to **(J)** upper panel 2 h after BrdU injection. **(L)** Corresponding to **(J)** lower panel 28 days after BrdU injection. Confocal images were taken with a Leica TCS SP5 II microscope. All graphs represent mean ± SEM. *p < 0.05, **p < 0.01, ***p < 0.001, significant relative to control; ns, difference not significant; hi, hilus; infra, infrapyramidal blade; mol, molecular layer; supra, suprapyramidal blade. Scale bars, 100 μm.

### Determination of Proliferating Progenitor Cells Using Ki-67 Immunolabeling

For quantification of hippocampal proliferating cell types 40 μm thick sagittal cryosections (1 in 12 series; from medial to lateral 2.725 mm to 0.225 mm, according to Allen Mouse Brain Atlas) from young adult WT and *P2ry13* KO mice spaced 160 μm apart were triple-immunostained for Ki-67, Tbr2 and DCX. Triple-immunostained z-stacks (1 ± 0.05 μm) were recorded with a Leica TCS SP5 II confocal laser scanning microscope and analyzed using Fiji (Schindelin et al., 2012) Cell Counter Plugin. In each section, three adjacent, non-overlapping regions of interest were imaged along the DG (capturing both infra- and suprapyramidal blades). Tbr2-stained cells, cells double-labeled for Tbr2/DCX, Tbr2/Ki67, DCX/Ki67, and cells triple-labeled for Tbr2/DCX/Ki67 were counted in individual sagittal cryosections and assigned to corresponding brain levels in WT and *P2ry13* KO mice (6 sections per animal, 7 animals per genotype). The total number of type 2a cells was determined by subtracting the number of Tbr2^+^/DCX^+^ cells from the total number of Tbr2^+^ cells. Proliferating type 2a cells were determined by subtracting the number of Tbr2^+^/DCX^+^/Ki67^+^cells from the number of Tbr2^+^/Ki67^+^ cells. DCX^+^/Ki67^+^ cells reflected the total number of DCX^+^- proliferating cells, including type 2b, and type 3 cells (immature neurons expressing DCX are postmitotic; compare Figure 3F).

### Quantification of Type 1 and DCX^+^ Cells

For quantification of subtypes of hippocampal progenitor cells, sagittal cryosections (1 in 12 series) from nestin-EGFP-expressing WT and *P2ry13* KO mice were double-immunostained for nestin EGFP and GFAP. Radial processes traversing the GCL positive for nestin-EGFP alone or double-labeled for nestin-EGFP and GFAP (type 1 cells) were counted in individual sagittal cryosections assigned to corresponding brain levels (4–6 sections per animal, *n* = 3–6). DCX^+^ cells (late type 2/type 3 cells and immature neurons) were counted in a separate series of experiments (six sections per animal, *n* = 7). Cleaved caspase-3^+^ cells were counted in the dentate GCL in serial coronal sections (14 sections per animal).

### Analysis of Microglial Numbers, Microglial Morphology and Dentate Gyrus Morphology

DAB-labeled Iba1^+^ microglia were counted in the hilus, the SGZ, the GCL and at the immediate border between the GCL and the inner molecular layer (ML; 1 in 12 series, four coronal sections per animal, *n* = 6) that were assigned to corresponding brain levels. Z-stacks (1 ± 0.05 μm) were recorded with a BioRevo BZ-9000 fluorescence microscope (Keyence, Neu-Isenburg, Germany) and analyzed using Fiji.

To identify alterations in hippocampal microglial morphology, structural parameters process area, process length, number of branch points and number process segments were determined using Imaris Software (Version 7.1.1). Fifty micrometer thick coronal sections of young adult WT and *P2ry13* KO mice (corresponding brain levels) were immunostained for Iba1 together with DAPI as described above. Data were acquired from seven WT and eight *P2ry13* KO mice and one section per animal corresponding to the stereotactic position −2355 to −2555 from Bregma (according to Allen Mouse Brain Atlas) was recorded using the Leica, TCS SP5 II confocal laser scanning microscope. In each section, three adjacent, non-overlapping regions of interest were imaged along the suprapyramidal blade starting from the crest. Maximum intensity projections of Z-series (distance 1 μm) were created using Imaris Surpass view and randomly selected microglial cells located in the SGZ were automatically traced and reworked using the Imaris Filament Tracer application. Values were averaged for each animal.

For analysis of changes in general DG morphology 40 μm thick serial sagittal cryosections (from medial to lateral 2.725 mm to 0.225 mm, according to Allen Mouse Brain Atlas) from WT and *P2ry13* KO mice spaced 160 μm apart were immunostained for NeuN. Based on NeuN immunofluorescence the area of the entire GCL was calculated in six sections per animal (assigned to corresponding brain levels, seven WT and six *P2ry13* KO mice) using the semiautomatic Contour Surface Tool in Imaris which allows to extract a 3D object by manually drawing the object contours on 2D slices.

### Quantification, General

To identify differences in the number of cells and cell types between WT and *P2ry13* KO mice different numbers of sections and in part different plains of sections were used in individual experiments. However, great care was taken to always analyze exactly corresponding brain levels for WT and *P2ry13* KO mice. This allowed the comparison of WT and *P2ry13* KO mice for each subset of experiments. Details of number of sections and plains of sections are provided in each Figure Legend.

### Statistical Analysis

All data are presented as mean ± SEM and statistical comparisons were assessed with the unpaired, two-sided Student’s *t*-test. *P* ≤ 0.05 was taken as significant.

## Results

### *P2ry13* mRNA Is Expressed in Microglia

For analyzing the functional role of the P2Y_13_R in hippocampal neurogenesis, we first determined its cellular distribution. Since none of the available antibodies against the P2Y_13_R yielded specific results when comparing *P2ry13* wild type (WT) and knockout (KO) mice, we applied FISH. Double labeling for the microglia/macrophage-specific ionized calcium binding adaptor molecule 1 (Iba1) shows that *P2ry13* mRNA is located in microglia (Figure 1A). Throughout the experiments specificity of the riboprobe used was monitored by applying the sense riboprobe and by analyzing the corresponding *P2ry13* KO mouse. No specific signals were obtained in sections from *P2ry13* KO sections or with the sense probe. The degree of colocalization between immunolabeling for Iba1 and *P2ry13* mRNA was 93.3 ± 0.8% (mean ± SEM; 777 *P2ry13* mRNA^+^ cells analyzed in three animals, see “Materials and Methods” section). We also performed double FISH for the microglia-expressed CX3C chemokine receptor 1 (*Cx3cr1*, Gemma and Bachstetter, 2013) and *P2ry13* and for Iba1 and *Cx3cr1* (Figure 1B). *P2ry13* mRNA was associated also with microglia in the cerebral cortex, cerebellum and hippocampal CA1 region and absent from NeuN^+^ neurons (Figures 2A,B).

We further analyzed cells of the hippocampal neurogenic pathway which can be identified by cellular markers for potential *P2ry13* expression (Figures 3A–F). These include type 1 cells extending a radial process into the GCL and expressing the GFAP (Figure 3A), type 1/type 2a cells expressing the transcription factor SRY (sex determining region Y)-box 2 (Sox2; Figure 3B), type 2b/type 3/immature neurons expressing doublecortin (DCX; Figure 3C), and finally mature neurons, expressing the neuronal nuclear antigen NeuN (Figure 3D; Kronenberg et al., 2003; Kempermann et al., 2015). *P2ry13* mRNA was neither associated with any of these cell types nor with normal GFAP-expressing astrocytes (Figure 3A). We furthermore excluded the expression of *P2ry13* mRNA in somatostatin- or parvalbumin-expressing interneurons (Figure 3E). These FISH-based data suggest that *in situ* the P2Y_13_R is expressed by microglia and neither expressed by cells of the hippocampal neurogenic pathway nor by neurons or astrocytes.

### Disrupting *P2ry13* Increases Progenitor Cell proliferation and New Neuron Formation

To define the functional role of the microglial P2Y_13_R in hippocampal neurogenesis we first studied progenitor cell proliferation and survival using the *P2ry13* null mouse model (Fabre et al., 2010). Several independent protocols were applied. Young adult WT and *P2ry13* KO mice received either one single intraperitoneal BrdU injection and were perfused 2 h after the BrdU pulse or 3 injections at 2 h interval and were perfused 3 days or 28 days later (Figure 4A). Figure 4B depicts the BrdU-labeled proliferative pool of cells in the DG. BrdU^+^ cells were almost exclusively observed in the SGZ. 2 h after the final pulse the number of BrdU^+^ cells was increased by 40% in *P2ry13* KO animals (WT 252.97 ± 13.57, *P2ry13* KO 354.43 ± 27.58, mean ± SEM; Figure 4C). The number of BrdU^+^ cells was still increased by 30% in *P2ry13* KO mice perfused 3 days after the final BrdU pulse (WT 506.00 ± 33.23, *P2ry13* KO 657.57 ± 44.03, mean ± SEM; Figure 4D) and it was duplicated after 28 days (WT 118.29 ± 13.42, *P2ry13* KO 241.00 ± 9.97, mean ± SEM; Figure 4E), implicating enhanced progenitor cell formation in *P2ry13* KO mice. The number of BrdU-labeled cells in the non-neurogenic hilar region was considerably lower than in the GCL and identical in WT and *P2ry13* KO animals (Figure 4F), demonstrating that the enhancement of cell proliferation was restricted to the DG. Neurogenesis was reported to be initially higher in the infrapyramidal than suprapyramidal blade (Snyder et al., 2012). We compared BrdU labeling in the supra- and infrapyramidal blades 2 h and 28 days after the final BrdU pulse. In both genotypes and at both time points the number of BrdU^+^ cells was higher in the supra- than in the infrapyramidal blade and in both blades the number of BrdU^+^ cells was higher in *P2ry13* KO mice (Figures 4G,H).

Hippocampal neurogenesis declines in rodents with increasing age (Kuhn et al., 1996). In an additional and independent series of experiments we investigated whether increased progenitor cell proliferation was maintained in aged *P2ry13* KO mice. In this case mice that had received three BrdU pulses were perfused 2 h after the final injection or—for analysis of long-term progenitor cell survival—mice received three daily BrdU pulses on three consecutive days and were perfused 28 days after the final pulse (Figure 4I). The number of BrdU^+^ cells was determined in the DG as depicted in Figure 4J. Similar to the results obtained with younger animals, the number of BrdU^+^ cells after 2 h, representing progenitor cell proliferation, was increased by 40% in *P2ry13* KO mice (WT 361.86 ± 29.19, *P2ry13* KO 506.93 ± 11.30, mean ± SEM; Figure 4K). But the number of BrdU^+^ cells after 28 days was only increased by 28% (WT 159.21 ± 11.12, *P2ry13* KO 203.79 ± 9.35, mean ± SEM; Figure 4L). This suggests that the relative increase in progenitor cell proliferation is maintained in aged *P2ry13* KO mice with reduced capacity for later integration into existing networks. Note, that absolute numbers of labeled cells cannot directly be compared between young (Figures 4C–E) and aged (Figures 4K,I) animals due to differences in the injection protocol and the number, plane, and thickness of sections analyzed (see “Materials and Methods” section).

**Figure 5:**
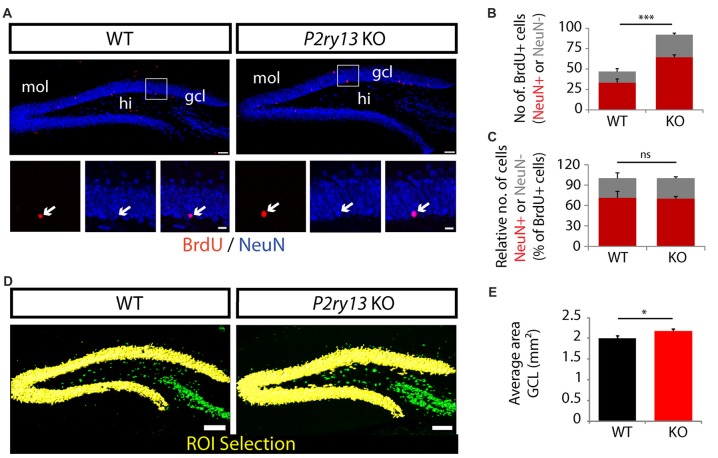
Increased neuron formation and thickening of the GCL in young adult *P2ry13* KO mice. **(A)** Immunostaining for BrdU (red) and NeuN (green) in the DG 28 days after BrdU labeling (compare Figure 4A). The lower panels represent close-up images of the regions indicated in the upper panels. **(B)** Total number of cells double-labeled for BrdU/NeuN (six corresponding sections per animal, *n* = 7). **(C)** Number of NeuN^+^ and NeuN^−^ cells as percentage of the number of BrdU^+^ cells (six corresponding sections per animal, *n* = 7; total number of BrdU^+^ cells counted in the GCL: 46.86 ± 4.43 in the WT and 92 ± 2.88 in the *P2ry13* KO). Red bars represent double-labeled cells, gray bars cells labeled for BrdU only. **(D)** Increased thickening of the GCL in *P2ry13* KO mice. **(E)** Quantification of the average area (six corresponding sections per animal) of the GCL in WT (*n* = 7) and *P2ry13* KO (*n* = 6) animals. Semiautomatic selection of the region of interest (ROI; yellow) was performed with the Contour Surface Tool in Bitplane Imaris in sections immunostained for NeuN. All graphs represent mean ± SEM. *p < 0.05, ***p < 0.001, significant relative to control, ns, difference not significant. gcl, granule cell layer; hi, hilus; mol, molecular layer. Scale bars, 100 μm in (**A**; upper panel) and **(D)**; 25 μm in **(A)** lower panel.

The data revealing increased numbers of BrdU-labeled cells after 28 days suggested that these BrdU^+^ cells may have matured into neurons. We next determined the number of BrdU^+^/NeuN^+^ neurons 28 days after BrdU injection in young adults (Figure 5A). As compared to WT mice the number of BrdU^+^/NeuN^+^ cells was increased by 93% in *P2ry13* KO mice (WT 33.43 ± 3.45, *P2ry13* KO 64.57 ± 1.85, mean ± SEM; Figure 5B). Importantly, the neurogenic capacity, the percentage of BrdU-labeled progenitor cells that finally differentiated into neurons, was identical between both genotypes (Figure 5C). In both cases 70% of the initial BrdU^+^ cells (100%) had become neurons (WT 70.63 ± 2.12, *P2ry13* KO 70.25 ± 1.26, mean ± SEM). This demonstrates that the increased pool of BrdU^+^ cells leads to increased neuron formation in *P2ry13* KO mice. The fate of the remaining 30% of BrdU^+^/NeuN^−^ cells has not been determined. These presumably represent macroglia (Steiner et al., 2004). Our data suggest that the relation of newly formed neurons to glial cells was not altered in *P2ry13* KO mice.

The constitutive increase in new neuron formation in *P2ry13* KO mice could result in increased neuron numbers in the DG reflected by increased thickness of the GCL. Following immunostaining for the neuronal marker NeuN (Figure 5D) we analyzed the area of the GCL semiautomatically using the Contour Surface Tool in Bitplane Imaris. As compared to WT controls the average area (mm^2^) of the GCL in young adults was increased by 9% in *P2ry13* KO animals (WT 2.00 ± 0.04, *P2ry13* KO 2.18 ± 0.06, mean ± SEM; Figure 5E). Taken together these data suggest that under basal conditions activation of the P2Y_13_R inhibits proliferation and new neuron formation, thereby acting as a brake on adult hippocampal neurogenesis.

### Disrupting *P2ry13* Does Not Affect Numbers of Neural Stem Cells but Expands Both Progenitor Cell Proliferation and the Pool of Progenitor Cells

**Figure 6:**
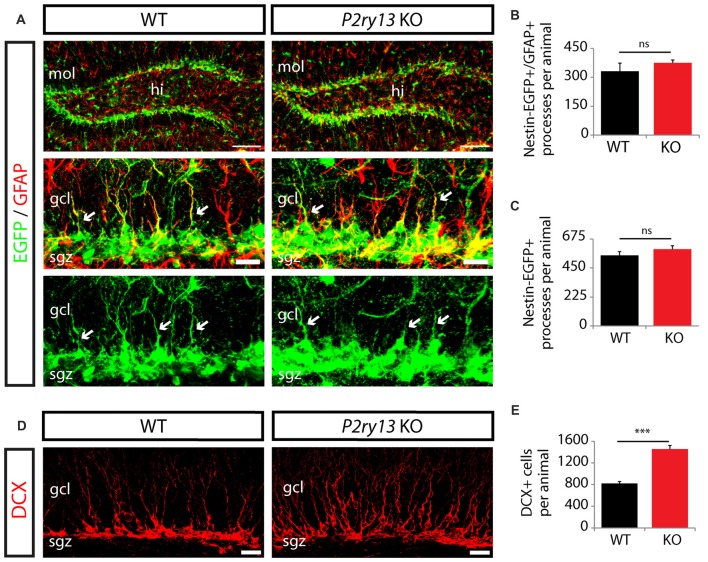
Unaltered neural stem cell numbers but enhanced expansion of DCX^+^ in *P2ry13* KO mice (young adults). **(A–C)** Radial processes were taken as a measure of cell number because of the substantial overlap between EGFP-filled cells in the SGL. **(A)** Immunostaining for nestin-EGFP (green) and GFAP (red) in the GCL of adult WT and *P2ry13* KO mice. The middle and lower panels represent close-up images of the upper panels. Radial cellular processes positive for nestin-EGFP and GFAP or for nestin-EGFP (arrows) and reaching into the GCL (both representing type 1 cells) were quantified in WT and *P2ry13* KO mice. **(B,C)** Total number of processes double-positive for nestin-EGFP/GFAP **(B)**, corresponding to middle panel in **(A)** or positive for nestin-EGFP alone **(C)**, corresponding to lower panel in **(A)** (four corresponding sections per animal, *n* = 3–6). **(D)** Immunostaining for DCX (red). **(E)** Total number of DCX^+^ cell bodies (six corresponding sections per animal, *n* = 7). All graphs represent mean ± SEM. ***p < 0.001, significant relative to control, ns, difference not significant. gcl, granule cell layer; hi, hilus; mol, molecular layer; sgz, subgranular zone. Scale bars, 100 μm in **(A)** (upper panel); 25 μm in **(D)**; 20 μm in **(A)** middle panel and corresponding lower panel.

Since a constitutive model of *P2ry13* disruption was used, the increase in the number of BrdU^+^ cells 2 h after BrdU labeling may have resulted from *a priori* increased numbers of neural stem cells in the *P2ry13* KO animals. We therefore compared the number of nestin-positive type 1 cells between WT and *P2ry13* KO animals (all young adults). To ease the identification of nestin-expressing cells, *P2ry13* KO mice were bred to mice expressing the enhanced green fluorescent protein (EGFP) under the control of the nestin promoter (nestin-EGFP mice). Due to the high contents of type 1 cells in EGFP, cell bodies in the SGZ could not be traced individually. We therefore counted the EGFP-positive radial processes reaching into the GCL as a measure for cell number. We quantified processes either double-positive for nestin-EGFP and GFAP or positive for nestin-EGFP alone (both representing processes of type 1 cells) in WT and *P2ry13* KO mice (Figure 6A). No difference was obtained between WT and *P2ry13* KO mice (Figures 6B,C), suggesting that the pool of type 1 cells was not enlarged in *P2ry13* KO mice. In an additional series of experiments, we determined the number of cell bodies immunopositive for DCX, which could be more easily discerned (Figures 6D,E). In contrast to type 1 cell processes, the number of DCX^+^ cells (representing late type 2 cells, type 3 cells and immature neuros) was increased by 77% (WT 821.57 ± 35.19, *P2ry13* KO 1457.33 ± 65.47, mean ± SEM) in *P2ry13* KO mice.

**Figure 7:**
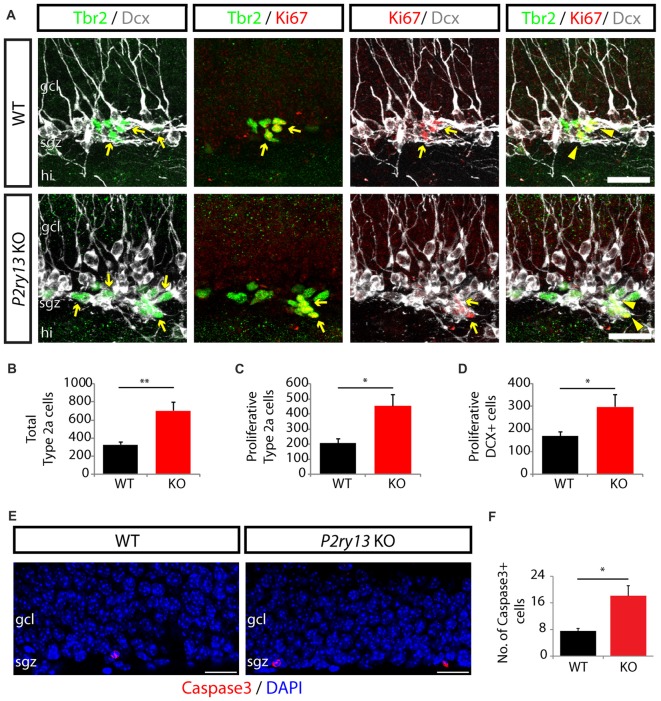
Increased progenitor cell proliferation and apoptosis in *P2ry13* KO mice (young adults). **(A–D)** Increase in number and proliferative capacity of type 2a cells and type 3 cells in *P2ry13* KO mice. **(A)** From left to right: Double labeling for Tbr2 (green) and DCX (white), Tbr2 (green) and Ki67 (red), Ki67 (red) and DCX (white) and triple labeling for Tbr2 (green), Ki67 (red) and DCX (white). Double-labeled cells are indicated by yellow arrows and triple-labeled cells by yellow arrowheads. **(B–D)** Resulting quantitative evaluation of the total number of type 2a cells **(B)**, the total number of proliferating type 2a cells **(C)**, and the total number of proliferating DCX^+^ cells **(D)** (six corresponding sections per animal, *n* = 7). **(E)** Immunolabeling for cleaved caspase-3 (red) in the GCL. DAPI staining is shown in blue. In both genotypes cleaved caspase-3^+^ cells were exclusively found in the SGZ and in low numbers. **(F)** Total number of caspase-3^+^ cells (14 corresponding sections per animal, *n* = 7). All graphs represent mean ± SEM. *p < 0.05, **p < 0.01, significant relative to control. gcl, granule cell layer; hi, hilus; sgz, subgranular zone. Scale bars, 25 μm in **(A)**; 20 μm in **(E)**.

We next investigated whether the increase in DCX^+^ cells and new neurons was due to maintained proliferation and which types of progenitor cells had changed their proliferative capacity, eventually resulting in increased new neuron formation (all young adults). Thus, we used Ki-67 labeling to identify proliferating cells and double and triple labeling for DCX and the transcription factor Tbr2 (T-box brain gene 2) as additional markers. DCX is expressed in type 2b and type 3 cells as well as in immature neurons (compare Figure 3F). The latter are, however, postmitotic (Kronenberg et al., 2003; Kempermann et al., 2015). Tbr2, a critical regulator of neurogenesis in the developing and adult DG, is specifically expressed in DG intermediate neuronal progenitors (comprising type 2a, type 2b and type 3 cells but not calbindin or NeuN-positive cells; Hodge et al., 2008). This allowed us to determine the number of proliferating type 2a cells (Tbr^+^/Ki67^+^ cells minus TBr^+^/DCX^+^/Ki67^+^ cells), the number of proliferating DCX^+^- cells (DCX^+^/Ki67^+^ cells), and the total number of type 2a cells (Tbr2^+^ cells minus Tbr2^+^/DCX^+^ cells) (Figures 7A–D). Our data revealed an increase, in *P2ry13* KO mice, in the number of proliferating type 2a cells and of proliferating DCX-positive cells of 119% (WT 207.00 ± 28.34, *P2ry13* KO 452.71 ± 77.39, mean ± SEM) and 76% (WT 168.86 ± 17.79, *P2ry13* KO 297.71 ± 54.02, mean ± SEM), respectively and in the total number type 2a cells of 115% (WT 326.14 ± 30.43, *P2ry13* KO 701.43 ± 96.40, mean ± SEM). These data imply that both type 2a cells and DCX-positive cells reveal similarly increased proliferation, resulting in constitutively increased total pools of both progenitor cell types in *P2ry13* KO mice.

Since microglia rapidly engulf and eliminate caspase-3^+^ apoptotic young hippocampal progenitor cells (Sierra et al., 2010) we compared the number of caspase-3^+^ cells in the DG between WT and *P2ry13* KO mice (young adults; Figure 7E). It was increased in the DG of *P2ry13* KO mice by 140% (WT 7.57 ± 0.72, *P2ry13* KO 18.14 ± 3.02, mean ± SEM; Figure 7F). However, the number of casapase3^+^ cells was low. The present data do not reveal the mechanisms underlying increased apoptotic cell death. Several scenarios are possible. Increased cell death could: (1) simply reflect increased progenitor cell proliferation in *P2ry13* KO mice; (2) the overall rate of apoptosis could be increased in *P2ry13* KO mice; and (3) decreased phagocytosis in WT microglia could result from delayed clearance time of apoptotic cells.

**Figure 8:**
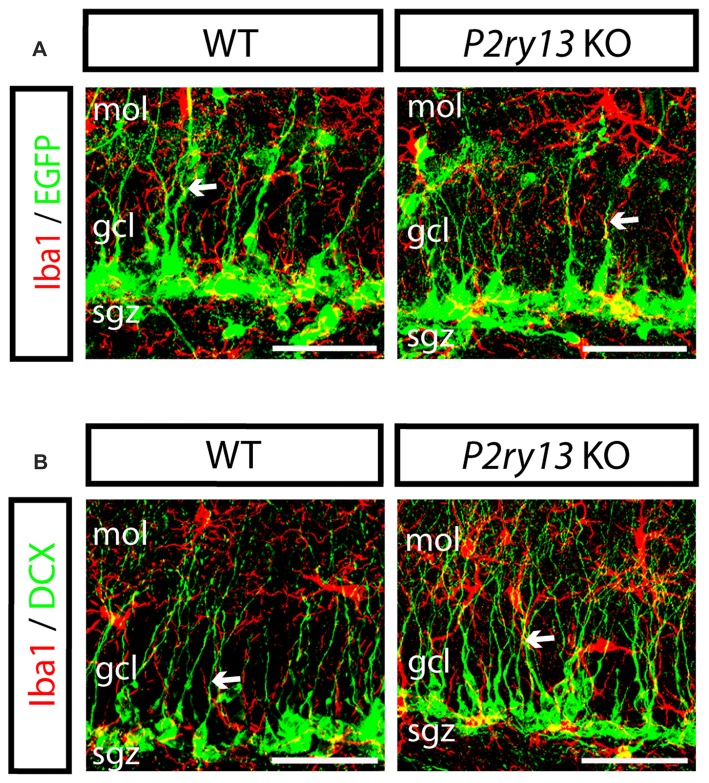
Close cellular interrelation between microglia and progenitor cells (young adults). **(A)** Double labeling of DG in WT and *P2ry13* KO for Iba1 (red) and nestin-EGFP (green); **(B)** Double labeling for Iba1 (red) and DCX (green). Microglia is intimately associated with neural precursor cells in the SGZ. Moreover, radial processes of microglial cells often run in parallel to nestin-EGFP- or DCX-positive radial processes and sometimes are intimately apposed (arrows). gcl, granule cell layer; mol, molecular layer; sgz, subgranular zone. Scale bars, 25 μm.

The results reported so far imply that microglia via the P2Y_13_R impacts neurogenesis in the DG. This requires close cellular contacts allowing chemical signal exchange. Thus, we found that in the SGZ, microglia is intimately associated with stem and progenitor cells (young adults). Moreover, via their radial processes microglia situated in the SGZ of either WT or *P2ry13* KO mice were often found running in parallel to or in close contact with radial processes of nestin-EGFP^+^ precursor cells or of DCX^+^ cells (Figures 8A,B).

### Structural Parameters but Not Numbers of Microglia Are Decreased in *P2ry13* KO Mice

**Figure 9:**
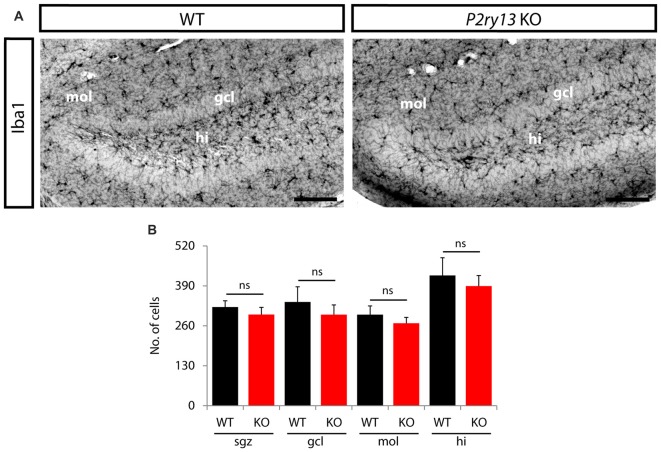
Disruption of *P2ry13* does not alter microglia number in the DG (young adults). **(A)** Iba1 immunoperoxidase-stained coronal sections of WT and *P2ry13* KO mice depicting the distribution of microglia in the DG and the penetration of microglial processes into the GCL. **(B)** Cell numbers were determined separately for the subgranular zone (sgz), granule cell layer (gcl), molecular layer (mol) and hilus (hi; four corresponding sections per animal, *n* = 6). All graphs represent mean ± SEM. ns, difference not significant. Scale bars, 100 μm.

**Figure 10:**
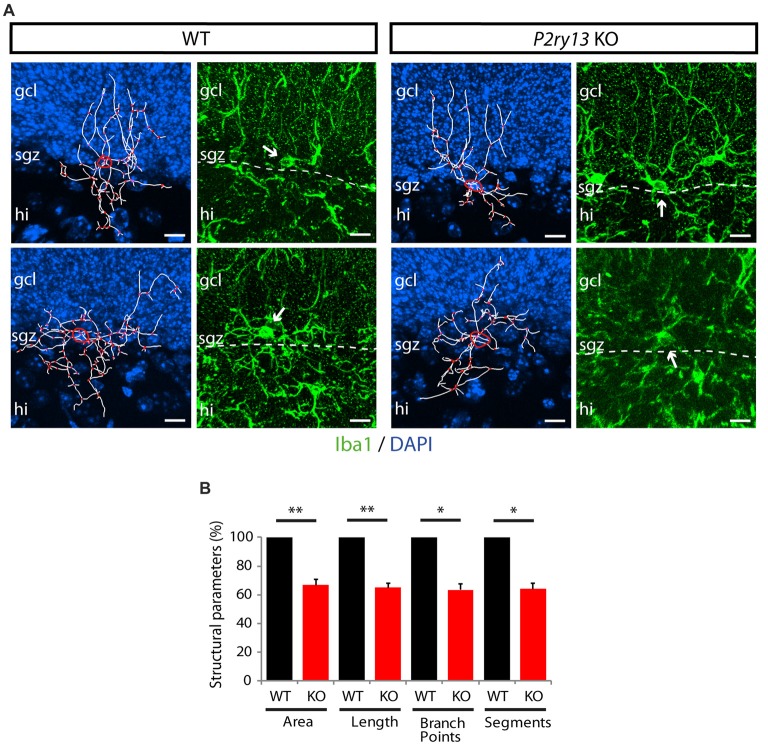
Reconstruction of individual microglial cells in the SGZ (young adults). **(A)** Cells from WT and *P2ry13* KO mice immunostained for Iba1 were used for Imaris Filament Tracer analysis. For each genotype a more polar cell type with processes reaching deep into the GCL (top) and a cell type with more radially distributed processes (bottom) is shown. Processes are depicted in white, branch points in red. The circumference of the cell body is indicated by a yellow line. DAPI staining of nuclei in blue. For each traced cell the original immunostaining for Iba1 is shown in the corresponding right panels. Note that 2D images do not depict all thin cellular processes. These can, however, be traced when using Imaris and moving 3D images in all directions during the tracing process. **(B)** Structural parameters for total process area (area), total process length (length), total number of process branch points (branch points) and total number of process segments (segments) are presented as relative values (± SEM). Absolute values (mean ± SEM) are for area (2,606.48 ± 121.35 μm^2^), for length (708.18 ± 30.11 μm), for branch points (51.38 ± 2.47), and for segments (111.75 ± 5.03). *p < 0.05, **p < 0.01. *n* = 7 for WT and *n* = 8 for *P2ry13* KO animals. Total number of traced cells: WT 40, *P2ry13* KO 50. hi, hilus; gcl, granule cell layer; sgz, subgranular zone. Scale bars, 20 μm.

The neurogenic impact of *P2ry13*-deficient microglia might be associated with altered microglial cell numbers or cell structure. Using Iba1 immunoperoxidase-stained sections we determined the number of microglial cells in the GCL, the SGZ, at the interface between the ML and GCL, and in the hilus (young adults; Figures 9A,B). No difference was observed between WT and *P2ry13* KO animals. Moreover, using Imaris Surpass view and the Imaris Filament Tracer application we determined structural parameters of microglial cells in the SGZ. Microglia revealed structural heterogeneity in the SGZ of both WT and *P2ry13* KO mice. While some cells project extensive processes into the GCL others display a more radial morphology (Figure 10A). The quantitative analysis revealed remarkable differences in structural parameters between WT and *P2ry13* KO mice (young adults; Figure 10B). In *P2ry13* KO mice the total process area was decreased by 33%, total process length by 35%, the number of process branch points by 37%, and the number of process segments by 36%. This demonstrates that ablation of *P2ry13* has a considerable impact on the structure of microglia in the hippocampal neurogenic niche.

## Discussion

**Figure 11:**
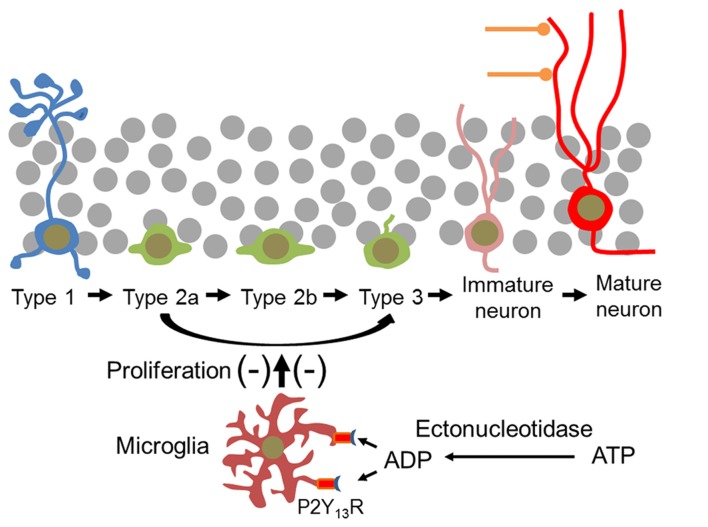
Microglial P2Y_13_Rs impair proliferation of neural progenitor cells and thus new neuron formation in the mouse DG. Type 1 cells are not affected. It is assumed that the effect of microglia is mediated by the P2Y_13_R- and ADP-induced release of extracellular signal molecules. In the hippocampal neurogenic niche the P2Y_13_R agonist ADP can be derived from extracellular ATP by ectonucleotidases which are associated with microglia itself and with type 1 cells and neural progenitor cells (Braun et al., 2000; Shukla et al., 2005).

Extracellular nucleotides play pivotal roles in the control of microglial functions. The present study assigns the ADP-activated P2Y_13_R to microglia in brain *in situ* and reveals that microglia located in the DG exhibit a specific structural profile enabling them to interact with both neural progenitor cells and maturing and mature granule neurons. Disrupting *P2ry13* impairs microglial structural complexity. It does not affect numbers of neural stem cells or microglia but increases progenitor cell proliferation and expands the pool of type 2a cells and DCX-expressing cells, and of newborn mature neurons (Figure 11). The data suggest that P2Y_13_R-expressing microglia function in constitutively attenuating hippocampal neurogenesis and play a significant role in the homeostatic control of neurogenesis. Following constitutive gene disruption developmental alterations in microglial function and neurogenesis cannot be excluded. Importantly, in the *P2ry13* KO mice neither the number of microglia nor of type 1 cells was altered, suggesting that these principal cell types were not affected.

### Disrupting *P2ry13* Impacts Microglial Structure in the Neurogenic Niche

Microglia have distinct region-dependent transcriptional profiles and their impact may vary between brain regions (Grabert et al., 2016). The activation pattern of microglia differs between neurogenic vs. non-neurogenic regions of the forebrain and between the two major neurogenic niches, the subventricular zone (SVZ) and the DG (Goings et al., 2006; Fourgeaud et al., 2016). In contrast to the ramified microglia in the cerebral cortex, microglia along the neurogenic SVZ, rostral migratory stream, and olfactory bulb regions have enlarged cell somata and relatively few and thick processes. Moreover, they display low expression of purinoceptors and lack ATP-elicitable chemotaxis (Ribeiro Xavier et al., 2015).

As observed previously (Shapiro et al., 2009; De Lucia et al., 2016) microglial cells vary in structure also in the hippocampus. At the dentate/hilus and at the dentate/ML interface they often extend radial processes penetrating or traversing the GCL. This implicates hippocampal microglial heterogeneity even though all structural phenotypes express the P2Y_13_R. We further show that microglial radial processes often run in parallel to and are in close contact with the radial processes of nestin-EGFP^+^ precursor cells and DCX^+^ neuroblasts, facilitating functional interaction. The reduction in microglial process extension and complexity determined in the SGZ of *P2ry13* KO mice suggests that the P2Y_13_R contributes to the structural development of microglia. Structural impairment of microglia may simply parallel but may also be causal for the observed increase in hippocampal neurogenesis in *P2ry13* KO mice. Interestingly, a recent study (Matyash et al., 2017) shows that constitutive deletion of NTPDase1, which would prevent hydrolysis of extracellular ATP to the P2Y_13_R agonist ADP at the surface of microglial cells (Braun et al., 2000), induces a reduction in structural parameters of cortical microglia similar to that observed in the SGZ of *P2ry13* KO mice in our study.

### Disrupting the Microglial *P2ry13* Increases New Neuron Formation

Disrupting *P2ry13* increases progenitor cell proliferation and the number of type 2a cells, DCX-positive cells, and new neurons. Of note, the neurogenic capacity, the relative amount of new neurons formed from BrdU^+^ precursors was identical in WT and *P2ry13* KO mice. These data suggest that disrupting *P2ry13* did not change the ratio of newborn neurons to other BrdU-labeled cells (presumably macroglia, Steiner et al., 2004) and that increased proliferation is a major factor for the increased neuron formation observed.

We further show that *in situ* and at the resolution of FISH, P2Y_13_R encoding mRNA is absent from astrocytes, neurons and progenitor cells. This is important since activation of granule neurons (Bruel-Jungerman et al., 2006) or of parvalbumin interneurons (Song et al., 2012) can promote neurogenesis. Our results correspond to RNA-sequencing transcriptome data obtained from acutely isolated cell populations from mouse cerebral cortex showing *P2ry13* expression selectively in microglia (Zhang et al., 2014). Previous reports on the *in vitro* expression of the P2Y_13_R in neurons (Miras-Portugal et al., 2016) or astrocytes (Carrasquero et al., 2009) are thus likely to reflect altered protein expression following cell culture. The influence of the P2Y_13_R on hippocampal progenitor cell proliferation and new neuron formation should therefore be exclusively mediated by microglia.

### ATP Can Be Released and Metabolized to ADP in the Neurogenic Niche

Several components of the purinergic signaling pathway have been identified in the hippocampal neurogenic niche. Hippocampal neural progenitor cells highly express nucleoside triphosphate diphosphohydrolase 2 (NTPDase2), a major ectonucleotidase of the hippocampal neurogenic niche, which hydrolyzes ATP and generates first ADP and eventually AMP (Shukla et al., 2005; Gampe et al., 2015). Deletion of the enzyme, which would prevent the extracellular production of the P2Y_13_R agonist ADP from released ATP, increases progenitor cell proliferation and expansion (Gampe et al., 2015). Moreover, extracellular ATP can be hydrolyzed to the P2Y_13_R agonist ADP by the microglia-associated NTPDase1 (Braun et al., 2000; Matyash et al., 2017). ATP can be released from hippocampal astrocytes (Cao et al., 2013) and from microglia (Imura et al., 2013). In addition, constitutive cellular ATP release (Lazarowski et al., 2011) could tonically activate microglial P2Y_13_Rs and attenuate neurogenesis. Microglial release of ATP could induce an autocrine loop (Murana et al., 2017) of ATP release and P2Y_13_R activation. The interstitial concentration of ATP in living rat brain (striatum) of 40 nM (Melani et al., 2012) would be—after hydrolysis to ADP—in the range of the EC_50_ value of the P2Y_13_R (Communi et al., 2001).

### Activation of the P2Y_13_R Can Induce a Considerable Number of Intracellular Signal Pathways and Microglial Cytokine Release

Results obtained from several cell lines and primary cultured cells reveal that P2Y_13_Rs can activate a considerable variety of intracellular signal pathways, which vary between cell types. P2Y_13_Rs couple to different G proteins (Gs/Gq). They trigger several intracellular pathways related to the activation of MAPKs (mitogen-activated protein kinases) and the phosphatidylinositol 3-kinase/Akt/glycogen synthase kinase 3 axis (reviewed in Pérez-Sen et al., 2017). Pathways include inhibition of adenylyl cyclase and stimulation of Erk1/2 (Communi et al., 2001) and CREB phosphorylation (Jacques et al., 2017), elevation of intracellular Ca^2+^ (Lyubchenko et al., 2011; Jacques et al., 2017), activation of the Nrf2/HO-1 (nuclear factor E2-related factor 2/hemoxigenase-1) axis protecting against oxidative stress-induced cell death (Espada et al., 2010), activation of RhoA and ROCK (Malaval et al., 2009), ROCK-dependent P38 MAK kinase (Tatsumi et al., 2015), or the ROCK/P38MAKP/NF-kb signaling pathway (Liu et al., 2017). In the pancreatic insulinoma-cell line MIN6c4, however, inhibition rather than activation of the P2Y_13_R enhanced ERK1/2, Akt and CREB phosphorylation, as well as cell proliferation (Tan et al., 2010). Moreover, P2Y_13_R activation induced gene expression and release of the inflammatory cytokines interleukin-1β (IL-1β), IL-6, and tumor necrosis factor α (TNF-α) in cultured dorsal horn microglia (Liu et al., 2017). P2Y_13_R activation in cultured dorsal spinal cord microglia increased intracellular Ca^2+^ concentrations (Zeng et al., 2014), which could in principle induce the release of various extracellular messengers and impact neurogenesis (Kuhn, 2015). This raises the possibility that P2Y_13_R-activated microglia releases messengers, which impact microglia itself as well as specific cell types contacted by microglia in the neurogenic niche and the GCL.

### Potential Mechanisms Impacting Microglia and Progenitor Cells Through Microglial P2Y_13_R Activation

The molecular mechanisms governed by the microglial P2Y_13_R in the control of microglial structure and hippocampal neurogenesis merit further investigation. Our data suggest that disrupting *P2ry13* impairs microglial structural complexity. The P2Y_13_R can couple to RhoA and Rock1 (Malaval et al., 2009). Activation of ROCK results in the stabilization of actin filaments and is involved in a considerable variety of functions involving cytoskeletal reorganization (Amano et al., 2010). A recent study on the role of spinal microglia in neuropathic pain in the rat (Tatsumi et al., 2015) revealed that intrathecal application of the slowly hydrolyzable P2Y_1_R/P2Y_12_R/P2Y_13_R agonist 2-methylthio-ADP significantly reduces the length of microglial processes and increases the number of primary processes, which is reversed by application of the ROCK inhibitor H1152. Whilst these data do not correspond to our findings in the SGL of the DG obtained with a constitutive P2Y_13_R deletion model, they highlight the potential of the P2Y_13_R to induce changes in microglial structure.

Inflammation is known to inhibit both basal and increased hippocampal neurogenesis in response to a brain insult, likely due to microglia-released cytokines (Ekdahl et al., 2003; Gonzalez-Perez et al., 2012; Sierra et al., 2014; de Miranda et al., 2017). However, increasing evidence suggests that pro-inflammatory cytokines have important signaling functions also in the normal adult brain (Bauer et al., 2007; York et al., 2017). Since P2Y_13_R activation induces microglial release of the pro-inflammatory mediators IL-1β, IL-6 and TNF-α (Liu et al., 2017), this opens up the possibility that these cytokines impact adult neurogenesis under normal conditions. For example, transgenic chronical astroglial IL-6 expression impaired neurogenesis in the hippocampal DG and reduced proliferation, survival and differentiation of BrdU-labeled neural progenitor cells, with gliogenesis progressing normally (Vallières et al., 2002; Campbell et al., 2014). Similarly, IL-1β has been shown to negatively affect various stages of hippocampal neurogenesis from the proliferation of neural precursor cells to their differentiation into neurons or astrocytes (O’Léime et al., 2017), and IL-1β is an essential mediator of the anti-neurogenic effects of stress (Koo and Duman, 2008). Also TNF-α has been implicated as a negative regulator of adult hippocampal neurogenesis (Iosif et al., 2006; Wang et al., 2018). While the complexity of the interactions of these cytokines is poorly understood one may speculate that the discontinuation of P2Y_13_R-mediated cytokine release in *P2ry13* KO mice may contribute to the observed increase in neurogenesis.

Our results suggest that the microglial P2Y_13_R receptor has a positive impact on microglial structure and a negative impact on adult hippocampal progenitor cell proliferation and new neuron formation. They implicate the microglial P2Y_13_R in the homeostasis of hippocampal neurogenesis. Selective P2Y_13_R antagonists could boost neurogenesis in pathological conditions associated with impaired hippocampal neurogenesis. Moreover, when analyzing nucleotide-controlled microglial functions, the microglial P2Y_13_R needs to be addressed equally well as the closely related P2Y_12_R.

## Author Contributions

JS and KG designed and performed experiments, interpreted results and edited the manuscript. MP and OT performed the *in situ* hybridization experiments and part of the immunohistochemical analysis, and OT and JS the Imaris image analysis. BR and J-MB generated the *P2ry13* KO mouse line. AA-P advised the project. HZ designed experiments and wrote the manuscript.

## Conflict of Interest Statement

The authors declare that the research was conducted in the absence of any commercial or financial relationships that could be construed as a potential conflict of interest.

## Footnotes

^1^http://mouse.brain-map.org/gene/show/50032
